# Phenotypes from cell-free DNA

**DOI:** 10.1098/rsob.200119

**Published:** 2020-09-02

**Authors:** Alexis Zukowski, Satyanarayan Rao, Srinivas Ramachandran

**Affiliations:** RNA Bioscience Initiative, and Department of Biochemistry and Molecular Genetics, University of Colorado School of Medicine, Mail Stop: 8101, 12801 East 17th Avenue L18–9102, Aurora, CO 80045, USA

**Keywords:** cell-free DNA, chromatin dynamics, subnucleosomes, cancer biomarker, DNA methylation

## Abstract

Cell-free DNA (cfDNA) has the potential to enable non-invasive detection of disease states and progression. Beyond its sequence, cfDNA also represents the nucleosomal landscape of cell(s)-of-origin and captures the dynamics of the epigenome. In this review, we highlight the emergence of cfDNA epigenomic methods that assess disease beyond the scope of mutant tumour genotyping. Detection of tumour mutations is the gold standard for sequencing methods in clinical oncology. However, limitations inherent to mutation targeting in cfDNA, and the possibilities of uncovering molecular mechanisms underlying disease, have made epigenomics of cfDNA an exciting alternative. We discuss the epigenomic information revealed by cfDNA, and how epigenomic methods exploit cfDNA to detect and characterize cancer. Future applications of cfDNA epigenomic methods to act complementarily and orthogonally to current clinical practices has the potential to transform cancer management and improve cancer patient outcomes.

## 1. Introduction

Blood is a minimally invasive source for tracking an individual's health status. Most known biomolecules found in blood, such as proteins, DNA, RNA, lipids, and metabolites inform us of some aspect of body function. However, using blood to diagnose and track cancer is still a major challenge. Minimally invasive diagnostics for cancer can greatly reduce pain and suffering of patients, and if cheaper than current methods, could be performed more often in the course of treatment to monitor disease state and inform clinical care. Genomic characterization of cancer either from biopsies or plasma cell-free DNA (cfDNA) has the added potential of providing personalized treatment options.

cfDNA is DNA bound to mononucleosomes and is found circulating extracellularly in the blood. cfDNA was first identified in 1948 [1] in the blood of patients; however, the hypothesis that cfDNA acts as a reflection of disease state arose from observations in 1977 [2]. This relationship between cfDNA and disease has been a subject of study ever since. There are multiple possible pathways for the release of DNA fragments from cells, including apoptosis, necrosis, and exosome secretion [3–6]. Processes that increase the release of cfDNA include disease, inflammation, tissue injury, and exercise [7,8]. In healthy individuals, haematopoietic maturation is a major contributor to the normal cfDNA pool. Lui *et al*. elegantly demonstrated that lymphoid/myeloid tissues are the major contributors to cfDNA by identifying Y-chromosome sequences in plasma of female recipients of bone marrow transplantations from male donors [9]. Multiple studies since then further confirmed that the lymphoid/myeloid tissues mainly contribute to the normal cfDNA pool [10–13]. Tumours, when present, also contribute to cfDNA. Thus, cfDNA is a molecular barcode of cells undergoing turnover and is an attractive target for clinical diagnostics and real-time monitoring of many cancers.

Provided that there are ways to distinguish cfDNA originating at the disease site from cfDNA produced during normal turnover, cfDNA could be used for diagnosis. The major focus of using cfDNA for cancer diagnosis has been the identification of a limited disease-specific panel of mutations. However, mutation detection has a significant limitation: that clonal haematopoiesis contributes to a significant fraction of mutations in cfDNA, including mutations in prominent cancer-associated genes like TP53 and DNMT3A [14]. Moreover, these mutations in cfDNA from the early stages of disease can be indistinguishable from mutations that arise at appreciable rates in healthy tissues [15,16]. This major obstacle has led to the exploration of other orthogonal information in cfDNA that could identify its tissue-of-origin and, in turn, disease states. Epigenomes reflect cellular identity and phenotype, and our ability to connect epigenomes to cellular identity could be used to infer disease phenotypes from cfDNA. Significantly, epigenome-based cfDNA approaches would be orthogonal and complementary to mutation-based cfDNA assays. In this review, we discuss epigenome features that can be characterized in cfDNA, and which provide information on the cfDNA tissue-of-origin.

## 2. Cell-free DNA contains a map of the chromatin state in the tissue-of-origin

The periodicity of cytoplasmic DNA released during red blood cell maturation in mouse fetal liver led to the hypothesis that there was a regular arrangement of protein protections on the genome [17,18]. This work preceded both the field of apoptosis and the biochemical characterization of the nucleosome and provided the first hint that cfDNA could be a map of chromatin structure. Protein-bound DNA lasts longer than naked DNA in serum [19], where nucleases are abundant, which suggests that cfDNA is probably double-stranded and protein-bound to inhibit degradation by endogenous nucleases in plasma. This is supported by the ability to detect nucleosomes in plasma by sandwich enzyme-linked immunosorbent assays (ELISA) and by chromatin immunoprecipitation (ChIP) [20–24].

**Figure 1.**
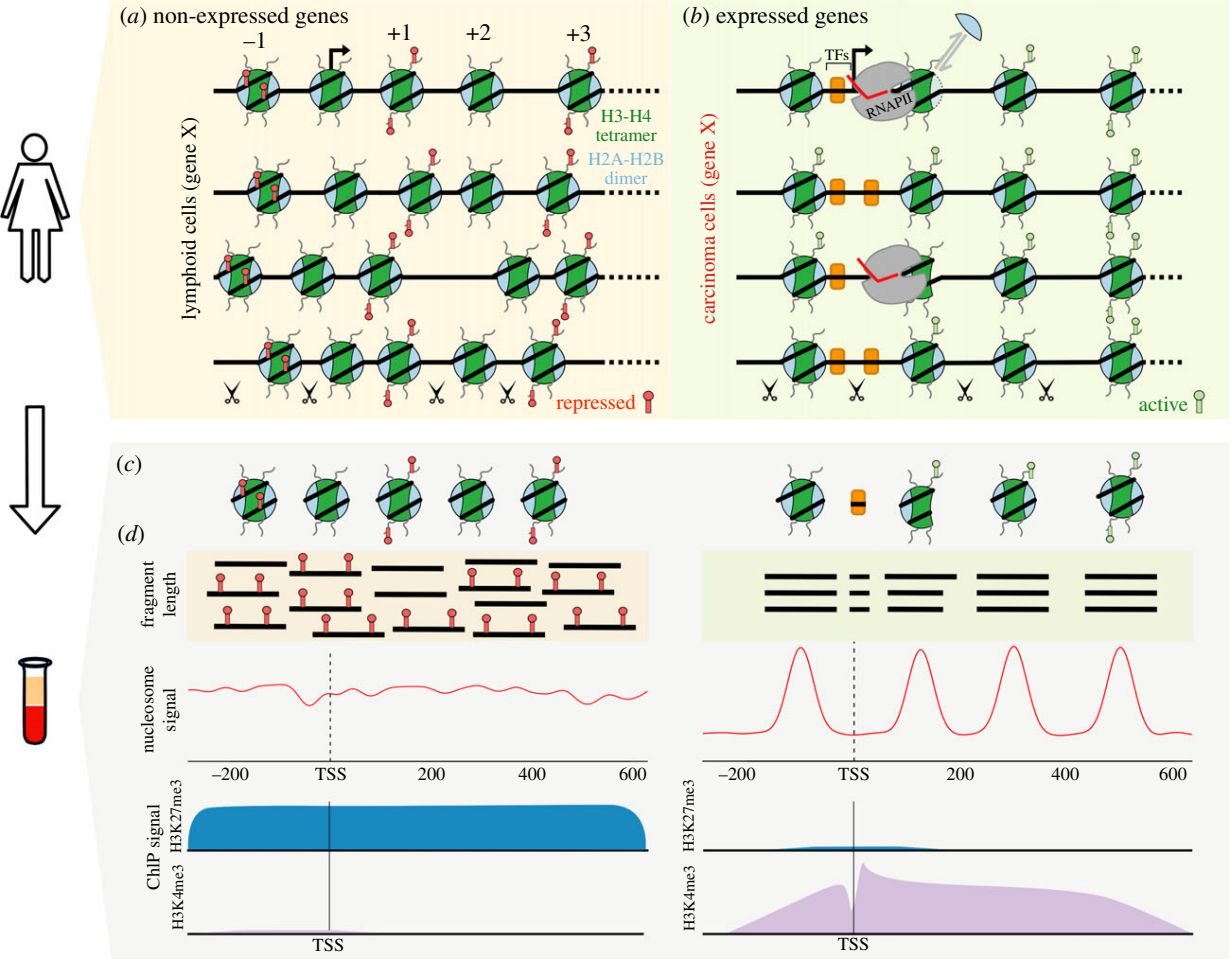
Cell-free DNA reflects the structural epigenomic information of the cell-of-origin. Schematic of the nucleosomal landscape differences of gene X when it is not expressed (left) and expressed (right) in different cell-types. (*a*) A non-expressed gene features promoter-proximal DNA methylation (red flags), methylation of nucleosomal histone tails (red flags, H3K27me3), nucleosome occlusion of the promoter (arrow), a lack of transcription factors (TFs) upstream of the promoter, and the absence of RNA polymerase II (RNAPII). (*b*) At expressed genes, nucleosomes are well-positioned, modifications like H3K4me3 (green flags) are present, RNAPII occupies the promoter, and TFs are bound upstream. At the +1 nucleosome during transcriptional elongation, RNAPII transiently breaks DNA-histone contacts allowing H2A-H2B dimers to exchange (light blue crescent). Nucleases, shown as scissors, are ubiquitously present and preferentially cleave accessible DNA. At gene X, when protein protections of DNA change during chromatin remodelling events, such as transcription, nuclease activity captures the different DNA length protections (see fragment lengths). (*c*) During cell-turnover, DNA-protein complexes are released into circulation. (*d*) Circulating DNA-protein complexes in plasma preserve the epigenomic features of the transcriptional status of the cell-of-origin. These features include fragment lengths (protein-DNA protections), DNA methylation, nucleosome positioning profiles, and nucleosome post-translational modifications. Note the preservation of these transcriptional and non-transcriptional DNA-protein species from in the body to plasma.

Apoptosis is one of the processes that could lead to genome fragmentation into small protein-DNA complexes that are found in circulation. Apoptosis involves the upregulation of nucleases that attack the cell's genome. Characteristic of this ‘attack' is DNA fragmentation that results in a laddering of repeat species less than 5 kb [5,25]. The repeating unit in laddering seen in apoptosis corresponds to approximately 150 bp, which parallels the length of DNA wrapped around a nucleosome. Therefore, plasma cfDNA, which is typically in the form of a mononucleosome, informs us about the chromatin structure of cells undergoing turnover. In other words, the ‘epigenome' of the tissue-of-origin can be measured from cfDNA (figure 1).

## 3. Structural epigenomics

It has long been known that chromatin structure in cells can be inferred from digestion patterns of nucleases that are sensitive to protein-DNA contacts [26]. Micrococcal nuclease (MNase) has been used to study chromatin structure for decades [27]. Understanding chromatin structure by sequencing fragments protected from MNase digestion has an unexpected parallel in cfDNA. The information derived from fragment length and genome location can inform us of biochemical activity at a genomic locus for MNase and cfDNA. For cfDNA, this can be used to infer transcriptional activity and tissue-of-origin. As we will demonstrate below, the length and genomic location of fragments protected from a nuclease can tell us the locus-specific distribution of nucleosomes, transcription factors (TF), and nucleosomal intermediates formed during transcription (figure 1). The ability to identify locus-specific structures of protein-DNA complexes from sequencing data, termed ‘structural epigenomics', is a general strategy applicable to a wide variety of datasets, including cfDNA sequencing datasets [11].

## 4. Nucleosome positions reflect genome function

Eukaryotic genomic DNA is packaged with nucleosomes like ‘beads on a string' and consecutive nucleosomes are separated by short linker DNA [28,29]. MNase preferentially degrades linker DNA and is inhibited when it encounters a protein-DNA contact [27,30]. A nucleosome protects 147 bp of DNA, a chromatosome (nucleosome with a linker histone bound) protects 167 bp of DNA, and TFs protect less than 50 bp of DNA. MNase has traditionally been used to map positions of whole nucleosomes in intact nuclei by purifying approximately 147 bp DNA after MNase digestion and subjecting this DNA to massively parallel short-read sequencing [31]. Nucleosome positioning impacts all biochemical processes that occur on the genome and consequently, knowing nucleosome positions enables us to predict biochemical activities that occurred in the cells that resulted in these protections [32]. A striking example of a distinct nucleosome organization is found in active genes. There is a depletion of nucleosomes at active promoters and nucleosomes are well-ordered upstream and downstream of active promoters. cfDNA contains information on nucleosome positioning and high-quality nucleosome maps were obtained from deep, whole-genome cfDNA sequencing from the plasma of healthy donors [12,33–35].

Nucleosome density inferred from cfDNA of healthy individuals had the same features as that of lymphoid cell lines: genes highly expressed in lymphoid cell lines featured nucleosome depletion at promoters and ordered nucleosome positions upstream and downstream of the promoter in cfDNA (figure 1). By contrast, genes that were not expressed in these cell lines showed significant nucleosome density over the promoters and lack of ordering over gene bodies in cfDNA [12,35]. These observations strongly validated the lymphoid/myeloid origin of cfDNA in healthy humans.

Quantitative scores were developed based on these striking observations to connect nucleosome profiles to gene activity in the cfDNA tissue-of-origin. Snyder *et al*. showed that stronger periodicity of nucleosomes in the gene body (the region between the transcription start site (TSS) and TSS+5000 bp) correlated with higher gene expression in lymphoid/myeloid tissues for healthy donors [12]. However, in cfDNA from donors with cancer, the correlation of nucleosome periodicity at gene bodies with gene expression of lymphoid/myeloid tissues was much weaker, suggesting other cell types contributing to the cfDNA pool. This was confirmed by the fact that the correlation of periodicity with gene expression of other cell types increased for donors with cancer [12]. Thus, nucleosome periodicity could inform the gene expression of the cfDNA tissue(s)-of-origin.

Ulz *et al*. noted that overall nucleosome density was lower in 2 kb upstream and downstream of the TSS (which they termed ‘2 K-TSS') and lowest at the promoter itself (usually termed the ‘nucleosome depleted region', NDR) in expressed genes in both MNase-seq of a lymphoblastoid cell line and the cfDNA sequencing data from healthy donors [35]. They developed a model that used the nucleosome occupancy at 2 K-TSS and NDR to predict gene expression. In a person with cancer, tumour-contributed cfDNA would have a depletion of nucleosomes at active genes, but this depletion may be masked by high nucleosome occupancy in cfDNA from lymphoid/myeloid tissues. Ulz *et al*. circumvented this problem by focusing on regions in the genome that featured copy-number gains in cfDNA of donors with cancer. These genomic regions would have a higher representation in tumour cfDNA if the tumour was the source of these copy number gains. Their simulations suggested that at least 75% of cfDNA at a given TSS must be released from tumour cells to infer expression status using their method. In the regions of copy number gain, they demonstrated significant changes in nucleosome occupancy at 2 K-TSS and NDR that correlated with increased expression of these genes in the tumour as assessed by RNA-seq of matched tumour biopsy samples. Thus, promoter and gene-body depletion of cfDNA fragments are indicative of gene activity in the tissue-of-origin of cfDNA.

## 5. Subnucleosomes in cell-free DNA inform expression state of genes

Transcription also results in the formation of subnucleosomes proximal to the promoter and identification of subnucleosomes could serve as a proxy for measuring gene activity. In eukaryotic cells, transcription occurs on a chromatin template. Because RNA polymerase II (RNAPII) needs to unwind the DNA strands to make RNA, every protein-DNA contact in its path needs to be disrupted. *In vitro*, nucleosomes present a substantial barrier to transcription elongation [36,37], resulting in specific stalls by RNAPII at sites of strong histone-DNA contacts. In cells, we can map the positions where RNAPII stalls with base pair-resolution maps of the 3′ ends of nascent transcripts [38–41]. These maps have shown that the first nucleosome downstream of TSS (+1 nucleosome) presents a strong barrier to RNAPII in cells.

The effect of RNAPII stalling on the +1 nucleosome could be understood by mapping intermediate nucleosome states during transcription elongation. With sequencing library protocols that capture all fragment lengths combined with paired-end sequencing, the full spectrum of fragments generated by MNase treatment of nuclei can be uncovered [11,42–44]. Fragments between 50 and 147 bp are too short to be protected by a whole nucleosome, but too long to be TF footprints. These intermediate-length protections represent a discrete loss of contacts asymmetrically from either side of the nucleosome as it unwraps during transcription and remodelling (figure 1). Correlating a base-pair resolution map of RNAPII with the high-resolution distribution of nucleosomal intermediates at the +1 nucleosome revealed that nucleosome unwrapping occurs in a stepwise manner—first, the contacts to the H2A-H2B dimer proximal to the promoter are lost. Then as RNAPII elongates through the nucleosome, contacts to the H2A-H2B dimer distal to the promoter are lost [11]. Cryo-electron microscopy of unwrapped nucleosomes and of RNAPII transcribing nucleosome templates yielded structures that matched the *in vivo* structures inferred from paired-end sequencing [45–47]. These observations highlight the ability of genomic sequencing of short DNA fragments to detect transcription-dependent substructures of nucleosomes in cells.

cfDNA is highly nicked and nicked DNA is lost in standard double-stranded library preparation protocols. Inspired by methods used in Neanderthal genome sequencing projects, Snyder *et al*. used a single-stranded library protocol (SSP) that captures both nicked and non-nicked fragments [12,48]. Surprisingly, they found an abundance of fragments less than nucleosomal size, ranging from approximately 40 bp onwards. These short fragments resemble subnucleosomes observed in MNase sequencing data from *Drosophila* cells [11]. When the subnucleosomal cfDNA fragments were mapped at +1 nucleosome positions of genes expressed in lymphoid/myeloid tissues, the same asymmetric unwrapping intermediates that were seen in *Drosophila* cells could also be observed in the cfDNA data [11]. Thus, it seems that the long residence time of RNAPII near the +1 nucleosome results in long-lived nucleosomal intermediate states that are mappable both by MNase, and by the endogenous nucleases that give rise to cfDNA.

In *Drosophila* cells, it was found that the amount of the subnucleosomal particles relative to nucleosomal particles at the +1 nucleosome of a gene correlated with the extent to which that gene was transcribed [11]. Therefore, it was hypothesized that the amount of these intermediates relative to whole nucleosomes (subnucleosome enrichment) at the +1 nucleosome of genes in cfDNA would correlate with the composite expression profile of cells giving rise to cfDNA. Indeed, in a healthy donor, cfDNA subnucleosome enrichment correlated much better with expression profiles of lymphoid/myeloid tissue types compared to other tissue types. However, in donors with cancer, the subnucleosome enrichment corresponding to lymphoid/myeloid tissues weakened substantially, enabling robust differentiation of healthy and cancer plasma cfDNA [11]. Thus, subnucleosomes in cfDNA represent relics of transcription in cfDNA tissues-of-origin that can inform us about the transcriptional programmes active at disease sites.

## 6. Chromatin structure correlations across megabases

The observation that nucleosome dynamics are reflected by shorter protections in cfDNA can be extended to much larger length scales than single nucleosomes, which allows maximal use of low depth cfDNA sequencing data. Using a machine learning model, Cristiano *et al*. were able to observe similar ‘fragmentation profiles' at megabase scales between cfDNA from healthy donors and the DNA released by MNase treatment of healthy lymphocytes [49]. Fragmentation profile is a measure similar to subnucleosome enrichment: a ratio of small fragments (100–150 bp) to large cfDNA fragments (151–220 bp). A significant difference in fragmentation profiles was observed between cfDNA from healthy donors and donors with cancer, enabling detection of cancer with low-depth whole-genome cfDNA sequencing. This method works presumably because fragmentation profiles at large length scales differentiate active domains of the chromosome from inactive domains. A tumour is expected to have significantly different active chromatin domains at the megabase scale compared to lymphoid/myeloid tissues.

A similar hypothesis is that the strength of correlation of cfDNA length profiles between genomic loci is proportional to the correlation of contacts made by the loci to the rest of the chromosome [50,51]. The correlation matrix of contact probability for a cell type can be obtained by Hi-C [52]. The Hi-C method involves crosslinking cells, fragmenting the genome into large pieces with the crosslinks intact, and then ligating the cross-linked fragments. Sequencing of the ligated contacts reveals chromatin contacts genome-wide. Regions of the genome with similar activity share similar contact profiles across the chromosome. Liu *et al*. showed that the strength of correlation of healthy cfDNA length profiles between different genomic regions is proportional to the strength of correlation of Hi-C contacts between different genomic regions as measured in lymphoblastoid cells. Furthermore, this correlation of cfDNA length profiles between different regions of a chromosome could be modelled as a combination of Hi-C maps of different tissue types to uncover tissue contributions to cfDNA in tumour samples with high (greater than 30%) tumour fractions [50]. Thus, cfDNA profiles could be used to infer chromosome contacts in the tissue(s)-of-origin.

## 7. Distinct cell-free DNA patterns at transcription factor binding sites

TF binding sites at promoters, enhancers, and insulators show enrichment of short protections (less than 50 bp) and depletion of nucleosomes in MNase-seq experiments [11,42,43,53–55]. Binding of many TFs also results in the ordering of nucleosomes around the TF binding sites [56]. Thus, protected TF binding sites represent a discrete class of genomic loci that show distinctive chromatin structural profiles in nuclease-protection assays. The enrichment of short fragments in cfDNA using SSP enabled Snyder *et al*. to observe similar enrichment of short protections and ordered nucleosome arrays at TF binding sites in cfDNA datasets from healthy plasma. They also observed an enrichment of short fragments in cfDNA at promoters that was proportional to expression levels of a lymphoid cell line, demonstrating the ability to possibly identify transcriptional complexes at a promoter from cfDNA [12].

Ulz *et al*. found that mononucleosome depletion observed in cfDNA at known TF-binding sites (TFBSs) is a good proxy for inferring TF binding [57]. This enables inference of TF binding from cfDNA sequenced using standard double stranded protocols that do not enrich for short fragments. They selected a set of TFs which are lineage-specific, for example, Androgen Receptor (prostate), Even-Skipped Homeobox 2 (colon), Forkhead box A1 (breast), and for each of them, profiled 1000 binding sites that were concordant across tissue samples [58]. Using plasma from patients with prostate, breast, and colon cancer, they showed that nucleosome depletion levels at binding sites of tumour-specific TFs are significantly higher compared to healthy cohorts and the reverse was true at binding sites for haematopoietic lineage-specific TFs such as LYL1, and EVI1. In addition, they also showed that nucleosome depletion levels at specific TFBSs were predictive of tumour subtypes. For instance, they found a significant reduction in nucleosome depletion at Androgen Receptor-binding sites when comparing samples from before and after a patient's prostate adenocarcinoma became androgen-independent. Thus, TF binding can be inferred from cfDNA either directly through short fragment protections when using SSP or indirectly via nucleosome depletion at TFBSs when using standard double-stranded library protocols and tissue-specific TFBSs enable identification of tissues that contribute to cfDNA.

## 8. Cell-free DNA fragment length

As we have discussed above, cfDNA length reflects the structure of chromatin in cells of origin and can be explicitly modelled as such. Beyond trying to understand cfDNA profiles in terms of chromatin structure, there have been several useful observations about the use of cfDNA length in biomarker development, for which we can only speculate the underlying molecular details. At its most general, the size of cfDNA fragments is used to delineate between baseline homeostasis and other biological phenomena that give rise to cfDNA. cfDNA has already been directly applied in the clinic for pre-natal testing. In plasma, fetal cfDNA is shorter than maternal cfDNA [13,59,60], which enables detection of aneuploidy or recessive disorders in the fetus non-invasively. Urine cfDNA features short periodic fragments that resemble subnucleosomes [51]. In individuals with cancer, circulating tumour DNA resides in the shorter cfDNA fraction [61–63]. The frequency of shorter, non-tumour cfDNAs is typically low and thus size selection for shorter fragments could dramatically increase the sensitivity of detection for mutant tumour cfDNA. Mutation targeting and identification of copy number variation and/or single copy number alterations is achieved with a higher sensitivity if the shorter cfDNA fraction is profiled [61,62]. This is a consequence of mutant tumour cfDNA being diluted less with non-tumour cfDNA in the shorter fraction. In the light of these observations, one prediction is that shorter cfDNA is a product of a specific mechanism that occurs at a higher frequency in fetal cells, cells that give rise to cfDNA in urine, and cancer cells relative to normal lymphoid/myeloid cell turnover.

## 9. Cell-free DNA chromatin immunoprecipitation uses cell type-specific genome-wide patterns of histone modifications to identify tissues of origin

Several studies in the early 2000s used ELISA to capture and calculate the concentration of nucleosomes in plasma [20,22–24,64,65]. Specifically, nucleosomes were captured in an antibody ‘sandwich', which used an antibody for a histone and an antibody for double-stranded DNA. This approach was an alternative means to assess cell death using plasma from cancer patients. In a notable study, cancer patients exhibited a statistically significant increase in nucleosome concentration compared to healthy individuals [20]. These findings suggest that nucleosome concentration could act as a biomarker for a disease state and/or discriminate between individuals with disease and those who are healthy. However, this application lacks specific genomic information to identify the tissue-of-origin and specify the disease state.

Beyond differences in overall nucleosome concentrations among physiological states, an intriguing question is if post-translational modifications (PTMs) of nucleosomal histones in plasma can be used to differentiate healthy individuals and those with disease (figure 1). PTMs of nucleosomal histones and DNA differ in euchromatic regions versus heterochromatic regions [66,67]. PTMs range from small chemical groups to larger proteins, such as ubiquitin, that are added to specific amino acids of proteins. For example, tri-methylation of histone 3 lysine 27 (H3K27me3) or H3K9me3 are found at heterochromatic regions where gene activity is repressed or ‘silent'. By contrast, H3K4me3 or H3K36me3 are associated with euchromatic regions where genes are active [68,69].

The link between cancer and chromatin regulation is well documented [70]. The majority of oncogenic mutations occur in genes of chromatin modifiers [71–73], which include a number of tumour-suppressor genes, such as SIRT1 [74]. Genomic instability is a hallmark of cancer and a consequence of alterations in heterochromatin that disrupt silencing [75,76]. Deligezer *et al*. analysed histone PTMs on circulating nucleosomes in plasma [22]. In light of the evidence for deregulation of repressive PTMs associated with gene silencing in cancer, histone methylation, specifically H3K9me1, was first assessed on circulating nucleosomes from myeloma patient plasma samples. Follow-up papers identified a reduction in repressive chromatin marks (H3K9me3, H4K20me3 and H3K27me3) in patients with colorectal cancer and metastatic prostate cancer [22,23]. However, these studies did not use high-throughput sequencing technology that would provide information on the genomic locations of histone PTMs; an aspect that is important to consider when delineating between different physiological states (i.e. disease and non-disease states) that produce changes in PTM levels in circulating nucleosomes.

A recent study sought to assess circulating post-translationally modified nucleosomes in a variety of physiological states using a combination of ChIP and next-generation sequencing [21]. The authors created a workflow to isolate modified circulating nucleosomes from plasma and performed high-throughput sequencing of the associated DNA (cfChIP-seq). Modified nucleosomes containing PTMs associated with active genes or *cis*-regulatory elements (i.e. enhancers) were specifically targeted: H3K4me1/2/3 and H3K36me3. H3K4me3 cfChIP-seq signal was used as a proxy for active genes and recapitulated earlier findings that cell-type specific gene activation programmes belonged to mainly blood lineages in a healthy individual. Furthermore, the cfChIP-seq signals reflected previously identified ChIP-seq signals for PTMs in these lineages.

This analysis was extended to profile patients with a variety of physiological insults, including surgery and cancer. cfChIP-seq detected differences in gene expression in individuals with cancer compared to healthy individuals. In response to surgical interventions, changes in tissue-specific cfChIP-signatures were detectable in the blood of patients supporting tissue-of-origin specificity. Combinations of PTMs were also assessed for complementary information to further profile tissue-type specific gene activity from non-coding regions. The authors observed changes in H3K4me2 (found at putative enhancer elements) and H3K36me3 (found within the body of transcribed genes) that correlated with differential gene activity unique to cancer tumours. Overall, the ability to identify the tissue-of-origin based on enrichment of circulating nucleosome PTMs associated with transcription at specific regions of the genome is exciting. cfChIP-sequencing provides an additional layer of information about an individual's underlying physiological state derived from the blood.

## 10. Cell-free DNA methylation patterns identify tissues of origin

Methylation of cytosines in the CpG dinucleotide context is associated with cellular identity [77] and can be identified on cfDNA [78,79]. Deamination of cytosine to uracil using sodium bisulfite or using an antibody specific to CpG methylation to pulldown methylated DNA are the two main methods used to map CpG methylation genome wide. Often promoter hypermethylation of a gene is associated with silencing [79]. DNA methylation profiles are stable and cell-type specific, and differentially methylated regions (DMRs) have been extensively used in quantification of subpopulations in DNA extracted from heterogeneous cell populations [80,81]. Accomando *et al*. showed that cell-types in leucocytes can be quantified using methylation status at as few as 20 CpG loci [82]. Many genomic locations exhibit highly cell-type specific DNA methylation, which has been instrumental in developing computational methods to delineate the contribution of tissues to cfDNA. CpG islands, a region 300–3000 bp in length and rich in G + C content, are usually hypomethylated, but aberrant methylation of these regions is associated with diseases like cancer [83]. Some CpG islands share high methylation levels across tumour types, while others are tumour specific [84]. Promoter hypermethylation of tumour suppressor genes is especially a striking feature of tumours particularly during carcinogenesis [85,86]. Targeted amplification of candidate promoter regions has been used to find that cfDNA from cancer patients exhibit hypermethylation in contrast to healthy donors [87–89]. Using methylated DNA immunoprecipitation (MeDIP-seq) optimized for low DNA amounts, Shen *et al*. detected numerous DMRs in cfDNA from individuals with cancer that are specific to tumour tissue-of-origin [90]. In particular, they showed that plasma DMRs in individuals with colorectal cancer closely matched solid tumour-derived DMRs. Thus, tissue-specific methylation patterns and hypermethylation of tumour suppressor genes can be used as signatures to detect cancer as well as identify cfDNA tissue(s)-of-origin [91].

## 11. Conclusion/future perspectives

**Figure 2.**
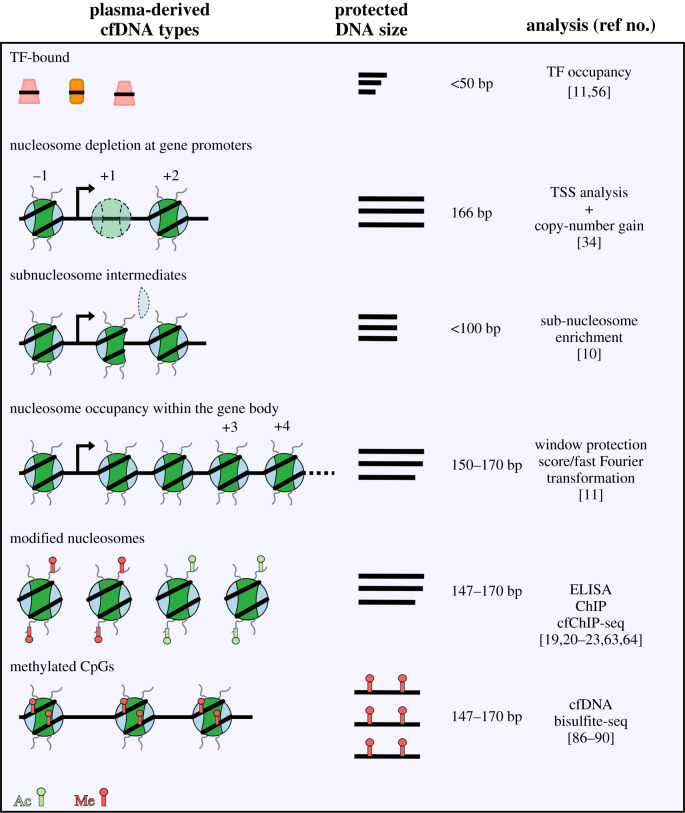
Clinical applications for cancer patients using epigenomic features of cell-free DNA.

The remarkable observation in 1970 that hinted at a relationship between cytoplasmic DNA and the chromatin structure of the cell-of-origin now underlies the conceptual paradigm of cfDNA epigenomics [18]. As there is an increasing awareness of the limitations associated with detecting and identifying diseases, such as cancer, solely based on mutant genotypes, the emergence of cfDNA approaches based on epigenomics provides a valuable alternative (figure 2). Chromatin remodellers, chromatin-modifying complexes and DNA methyltransferases are frequently dysregulated in cancer highlighting the relationship between chromatin regulation and oncogenesis [92–97]. Furthermore, simulations show that using hundreds of features throughout the genome (which would be the case for epigenomic features) improves the limit of detection of disease states from cfDNA compared to a few features (which would be the case when looking for tumour mutations) [49,90]. Combining epigenomic assessments with mutation-based targeting may better guide personalized treatment of cancer patients rather than solely relying on one method.

Early detection of cancer still remains a challenge for all cfDNA-based methods. An early cancer diagnosis increases a patient's chance for curative treatment. However, tumour shedding of DNA into the blood is at a lower concentration in early stages than what is observed in advanced cancer stages. Most methods described above have been used on samples of advanced cancer patients indicating a much-needed effort to examine their use and sensitivity for early detection. However, significant in-roads are being made. A recent multi-centre, randomized study used cfDNA methylation analysis to prospectively assess the largest cohort (*n* = 6689, 4207 healthy and 2482 cancer) to date for early detection and localization of more than 50 types of cancers [91]. With 99.3% specificity, the sensitivity increased as cancer stage increased (Stage I: 18%—Stage IV: 93%), with tissue-of-origin predicting cancer in 96% of samples with an accuracy of 93%. These are promising results for the future development of a cancer screening test for the general population. With improvements over time, cfDNA epigenomic methods described in this review can be combined with current clinical practices to improve detection of cancer in patients.

Obtaining molecular signatures of cancer based on epigenomic features highlights the variety of information contained in the blood of an individual. The studies we reviewed here represent an orthogonal network of disease-specific epigenomic information that is rich for exploitation. Combined with current clinical molecular diagnostics, the timely emergence of epigenomic cfDNA methods herald a chance to revolutionize disease management, specifically cancer. One can envision the successful implementation of these approaches to help guide personalized medicine at different stages of disease beginning with diagnosis and throughout the course of treatment. Beyond biomarkers, diverse epigenomic cfDNA methodologies also provide the opportunity to directly investigate the molecular mechanisms underlying pathology in humans.

## Data accessibility

This article has no additional data.
